# Extremely Long Spinal Cord Infarction

**DOI:** 10.31662/jmaj.2023-0042

**Published:** 2023-05-22

**Authors:** Daiki Yashita, Akihito Hao, Naohiro Uchio, Hideyuki Matsumoto

**Affiliations:** 1 Department of Neurology, Mitsui Memorial Hospital, Tokyo, Japan

**Keywords:** spinal cord infarction, posterior spinal artery syndrome, longitudinal lesion

The patient was a 78-year-old woman who developed extremely long spinal cord infarction over one month. She had a history of hypertension and obesity. Neurological examinations showed complete paraplegia, diminished superficial sensation at the T2-L5 dermatome levels, diminished deep sensation of legs, dysuria, dyschezia, and positive Babinski signs. Laboratory, physiological, and imaging examinations did not show any findings suggesting vascular malformation, myelitis, embolism, or arterial dissection. On spinal magnetic resonance imaging, T2- and diffusion-weighted imaging showed hyperintensity lesions in the posterior and bilateral parts of the T2-L3 vertebral levels of the spinal cord (Figure 1, arrowheads). The length was equal to 13 vertebral bodies.

**Figure 1.**
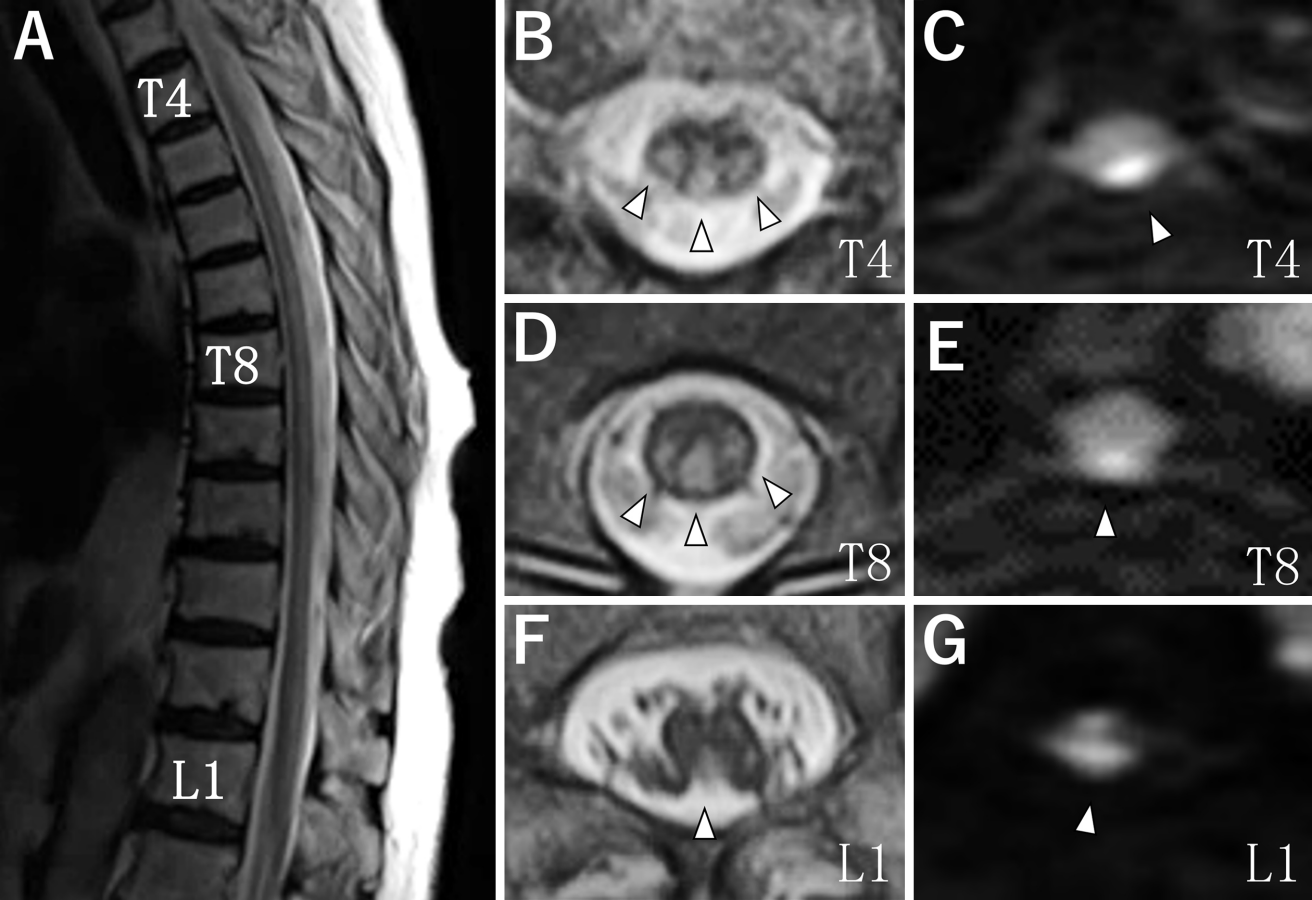
Spinal magnetic resonance imaging. Sagittal T2-weighted imaging (T2WI) showed longitudinal hyperintensity from vertebral level T4 to the conus medullaris of the spinal cord (A). Axial images showed hyperintensity in the posterior part of the spinal cord on T2WI and diffusion-weighted imaging (DWI) (B-G arrowheads). T2WI (B, D, F). DWI (C, E, G). The vertebral levels of each image are as follows: T4 (B, C), T8 (D, E), and L1 (F, G).

The average length of spinal cord infarction is reported to be approximately three vertebral bodies ^(1)^. Long spinal cord infarction has been rarely reported in posterior spinal artery syndrome ^(2), (3)^. Its rarity has been explained by abundant anastomosis between paired posterior spinal arteries ^(4)^. Recently, however, the great posterior radiculomedullary arteries longitudinally feeding the dorsal part of the spinal cord, similar to the artery of Adamkiewicz for the ventral part, has been identified in 72% of human spinal cord specimens: one unilateral artery 48%, two bilateral arteries 22%, and three unilateral arteries 2% ^(5)^. We speculate that the single, well-developed, great posterior radiculomedullary artery would be occluded by atherosclerosis. Based on the above discussion, we should consider spinal cord infarction, including posterior spinal artery syndrome, even when spinal cord lesions are extremely long.

## Article Information

### Conflicts of Interest

None

### Author Contributions

DY wrote the first manuscript. AH, NU, and HM edited and approved the final manuscript.

### Approval by Institutional Review Board (IRB)

IRB approval was not required for this study.

### Informed Consent

Informed consent was obtained from the patient.
